# Decreased physical activity with subjective pleasure is associated with avoidance behaviors

**DOI:** 10.1038/s41598-022-06563-3

**Published:** 2022-02-18

**Authors:** Fumi Kagawa, Satoshi Yokoyama, Masahiro Takamura, Koki Takagaki, Yuki Mitsuyama, Ayaka Shimizu, Ran Jinnin, Hirotaka Ihara, Akiko Kurata, Go Okada, Yasumasa Okamoto

**Affiliations:** 1 Department of Psychiatry and Neurosciences, Graduate School of Biomedical and Health Science, Hiroshima University, Hiroshima, Japan; 2 Department of Neurology, Shimane University, Shimane, Japan; 3 Brain, Mind and KANSEI Sciences Research Center, Hiroshima University, Hiroshima, Japan; 4 Health Service Center, Hiroshima University, Hiroshima, Japan

**Keywords:** Psychology, Health care

## Abstract

The main hypothesis for the relation between physical activity and mental health is that autonomous motivation, such as subjective pleasure for the activity, plays an important role. However, no report has described empirical research designed to examine the role of subjective pleasure in the relation between objectively measured physical activity and psychological indexes. We used accelerometers to collect data indicating participants' physical activity intensity during a week. Participants recorded their subjective pleasure of activity per hour. In 69% of them, the individual correlation coefficients between physical activity and pleasure in an hour (an index of Physical Activity-Pleasure; PA-PL) were positive (*r* = 0.22, 95%Cl = [0.11–0.38]), indicating that pleasant sensations increased concomitantly with increasing physical activity. Conversely, 31% participants exhibited negative values of PA-PL, which means that the increase in physical activity had the opposite effect, decreasing pleasure. Multiple linear regression analysis showed that avoidance/rumination behaviors decreased significantly with increased PA-PL (β = −6.82, 95%CI: [−13.27 to −0.38], *p* < .05). These results indicate that subjective pleasure attached to the PA is more important than the PA amount for reducing depressive behavior.

## Introduction

Many reports have described that increased physical activity engenders decreased negative emotions, increased positive emotions, and improvement of mental health including depression and anxiety^1,2^ and self-esteem^3^ in healthy adults. A recent large study conducted in the US, however, showed that although physical activity was associated with mental health improvement during the prior month, greater amounts of physical activity did not always exhibit better effects^1^. A study with an intervention to increase physical activity for 5 weeks demonstrated that a low-to-moderate intensity exercise intervention program provided greater improvement of depression and anxiety than the high-intensity program^4^, which also indicated that the physical activity and its intensity are not simply correlated with mental health.

A meta-analysis of domain-specific physical activity^5^ showed that, although global physical activity was not associated consistently with mental health, leisure-time physical activity and transport physical activity had a positive correlation with mental health. However, the positive effect of transport physical activity in young people who had to walk was less than that of adults. Work-related physical activity of manual laborers reportedly had a more negative effect on mental health than it had on office workers^6^. Results indicated that personal autonomous motivation might intervene between physical activity and improvement in mental health^7^.

Using a method of concept analysis from the literature, Palmer et al.^7^ found that a desire to do exercise which one has because the behavior is “fun” and “enjoyable” is autonomous motivation. Lewinson et al.^8^ reported that increased physical activity leading to individuals’ pleasure and enjoyment has effects of improving depressive mood and behaviors such as rumination and avoidance. However, no report of the relevant literature has examined the association between subjective pleasure related to autonomous motivation and physical activity measured objectively using accelerometers and other devices. Moreover, no report in the relevant literature has explored how the association between physical activity and subjective in daily life affects the characteristics of depression-related behaviors such as avoidance patterns in individuals.

For this study, we examined the association between physical activity and subjective pleasure by objective measurement of amounts of physical activity using an accelerometer and subjective measurement of pleasure using an activity record. We also investigated how the relation between physical activity and subjective pleasure affects individual behavioral characteristics, especially depression-related behaviors such as avoidance patterns.

## Results

### Background and psychological and behavioral characteristics of participants

Table 1 presents psychological and behavioral characteristics of the participants. Participants were 37 men and 21 women, with mean age of 21.78 ± 1.63. For physical activity and subjective pleasure, the total score for 1 week and the mean of the hourly scores were shown.

**Table 1:** Psychological and behavioral characteristics of participants.

Variable	Mean ± SD
BDI-II	5.21 ± 5.56
BADS-total	107.79 ± 17.5
BADS-AC	23.45 ± 7.42
BADS-AR	12.62 ± 7.93
BADS-WS	8.21 ± 4.99
BADS-SI	2.83 ± 3.76
Physical activity (sum)	12332.54 ± 939.33
Physical activity (hourly average)	76.65 ± 3.85
Pleasure (sum)	6835.86 ± 3179.26
Pleasure (hourly average)	41.64 ± 19.22

*BDI-II* Beck Depression Inventory, *BADS* Behavioral Activation for Depression Scale, *BADS-AC* activation subscale, *BADS-AR* avoidance/rumination subscale, *BADS-WS* work/school impairment subscale, *BADS-SI* social impairment subscale.

### Association between physical activity amount and behavior characteristics

Table 2 presents partial correlation coefficients between Physical activity/Pleasure and behavior characteristics while controlling for effects of the variables of BDI-II, age, and gender.

**Table 2:** Partial correlations between physical activity/pleasure and behavior characteristics.

	BADS-total	BADS-AC	BADS-AR	BADS-WS	BADS-SI
Physical activity (sum)	−0.032	−0.054	0.029	−0.048	0.028
Physical activity (hourly average)	0.009	0.155	0.199	−0.038	−0.018
Pleasure (sum)	0.052	0.260	0.157	−0.048	0.125
Pleasure (hourly average)	0.059	0.268	0.159	−0.056	0.120

*BADS* Behavioral Activation for Depression Scale, *BADS-AC* activation subscale, *BADS-AR* avoidance/rumination subscale, *BADS-WS* work/school impairment subscale, *BADS-SI* social impairment subscale.

The physical activities themselves and the sum score of pleasure had no significant correlation with the behavioral characteristics, but the mean of the hourly pleasure score showed positive correlation with BADS-AC (*r* = 0.268, *p* < 0.05). This result indicates that the hourly pleasure level is associated with the level of behavioral activation in BADS.

### Association between physical activity and subjectively measured pleasure during the same period: results found for the index of Physical Activity-Pleasure

**Figure 1:**
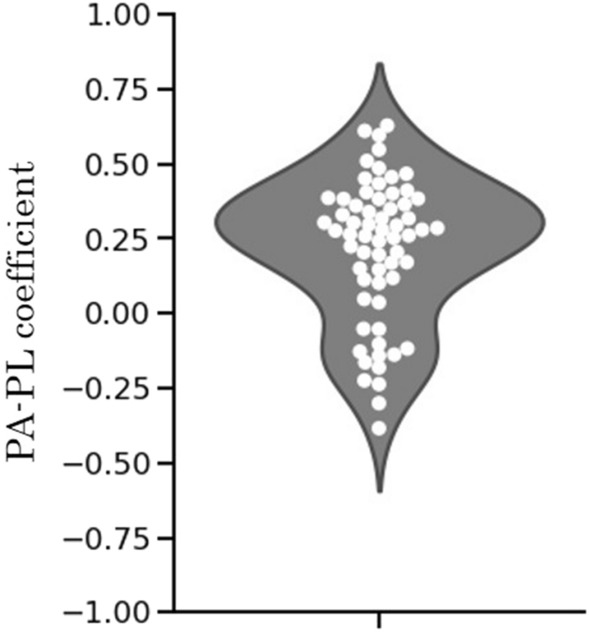
Correlation coefficients between physical activity and pleasure (PA-PL). White dots represent the PA-PL values of the respective participants. Each point is adjusted (along the x-axis) so that they do not overlap. The scatterplot along the x-axis does not reflect the magnitude of any value. The gray color shows the kernel density estimation.

Figure 1 shows individual correlation coefficients between physical activity and pleasure on an hourly basis (an index of Physical Activity-Pleasure; PA-PL) for all participants.

The PA-PL index, on average, was found to have positive correlation (*r* = 0.22, 95%Cl = [0.11–0.38]), which indicates that the pleasure increased concomitantly with increased physical activity.

**Figure 2:**
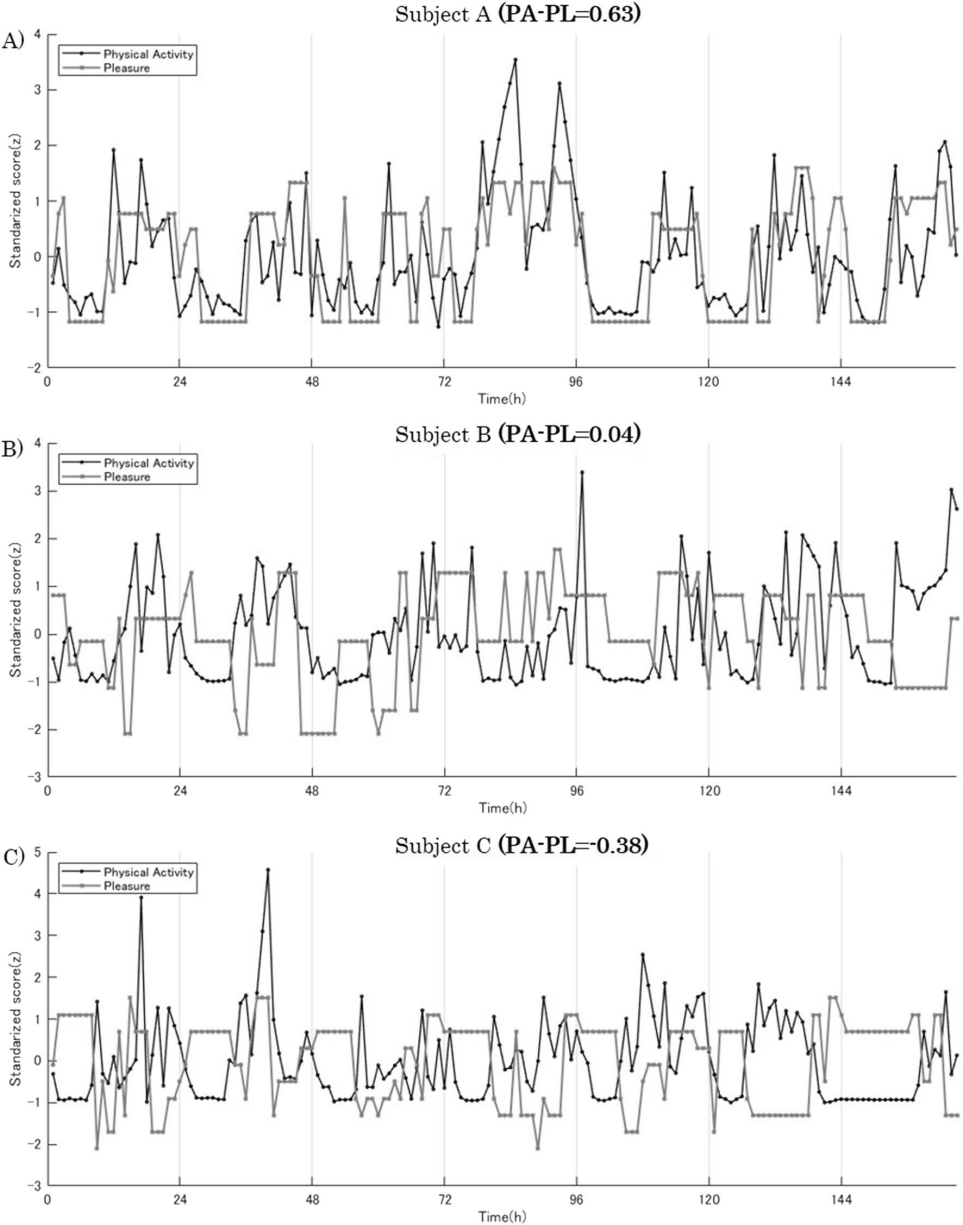
Time-series plot of physical activity and pleasure in 7 days for representative three-samples. **(A)** Plot for participants with the highest PA-PL (PA-PL = 0.63). **(B)** Plot of participants with the PA-PL nearest to zero (PA-PL = 0.04). **(C)** Plot of participants with the lowest PA-PL (PA-PL = −0.38). Ranges of the score differ considerably between physical activity and pleasure. Each score was normalized for the *y*-axis, with zero corresponding to the average.

The individual data, however, indicate that not all participants were found to have a positive correlation coefficient between the amount of physical activity and pleasure. Although 69% (40 out of 58; 32 out of 40 had a significant positive correlation at the 5% significance level) participants were found to have a positive correlation (e.g., Fig. 2A participant, Fig. 2B participant), 31% of participants (18 out of 58; 11 out of 18 had a significant and positive correlation at the 5% significance level) were found to have negative correlation (e.g., Fig. 2C participant). The findings indicate that pleasure correlates positively to increased physical activities in more than two thirds of the participants, but negatively in about one-third of them, indicating that the increase in physical activities had the opposite effect of decreasing pleasure. These results suggest that individual differences exist in terms of correlation between increased duration of physical activities and experiences of pleasure.

### Association between PA-PL index and behavior characteristics

**Figure 3:**
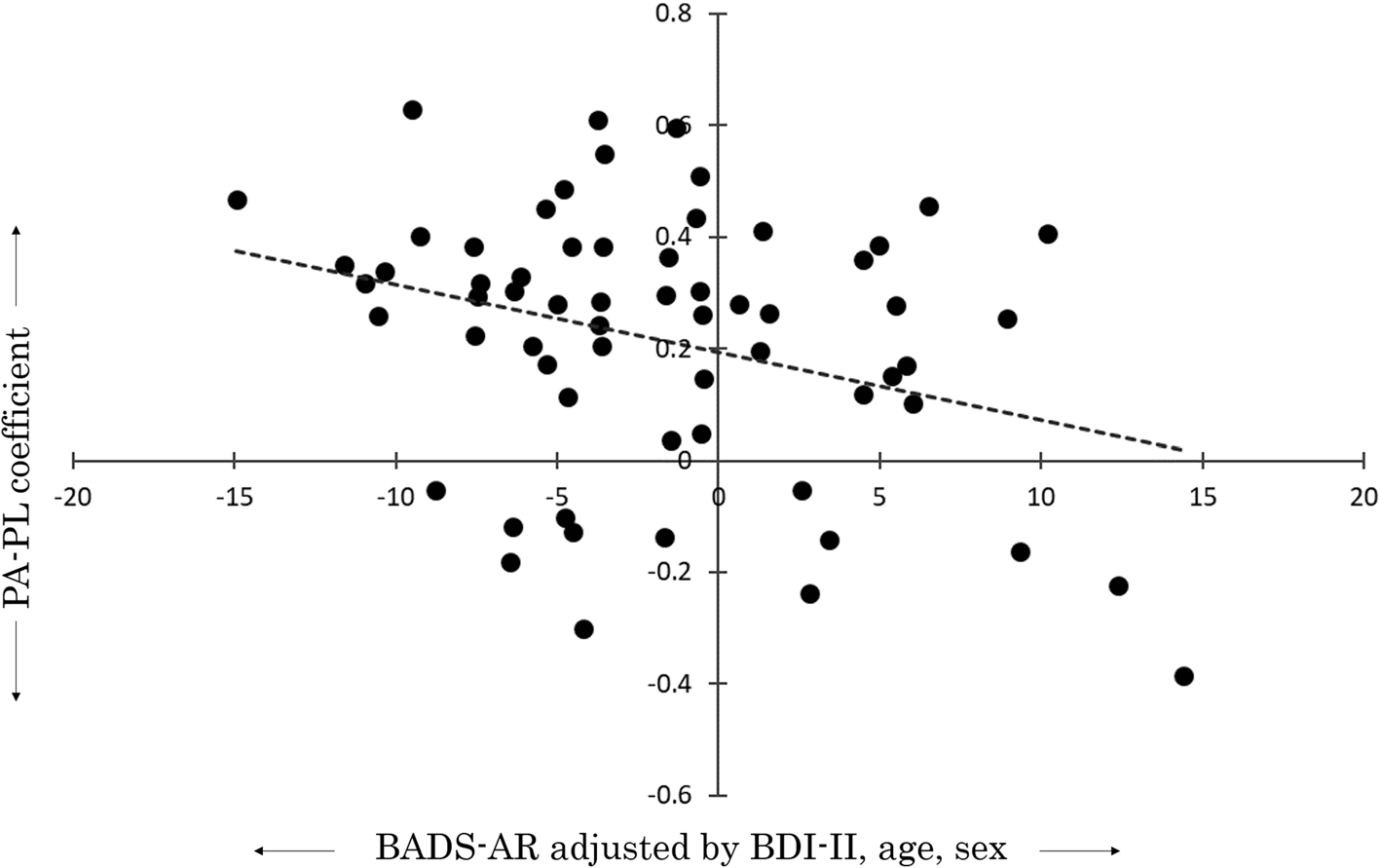
Scatter plot of PA-PL coefficients for BADS-AR. The avoidance/rumination subscale (BADS-AR) on the x-axis is adjusted by all covariates.

Multiple linear regression analysis using the scores of BADS subscales as dependent variables showed that BADS-AR increased significantly with decreased PA-PL (β = −6.82, 95%CI: [−13.27 to −0.38], *p* < 0.05) (Fig. 3). In fact, PA-PL had no significant effect on BAD-AC, WS, or SI because of the strong effect of BDI-II (AC: β = 1.09, 95%CI: [−6.72 to 8.90], n.s.; WS: β = −4.31, 95%CI: [−9.27 to 0.64], n.s.; SI: β = −3.53, 95%CI: [−7.06 to 0.01], n.s.) (Table 3). From the simple correlation analysis, the subscale scores including BADS-AR showed no significant correlation coefficient with PA-PL (Supplementary Table S1). The effect of BDI-II on BADS-AR is inferred as extremely strong. In addition, even if they were adversely affected by the same severity of depressive symptoms, participants found to have more negative correlation between physical activity and pleasure exhibited a stronger tendency for avoidance patterns. The VIF values observed among the explanatory variables were all too small for multicollinearity concerns: all VIFs were less than 1.12.

**Table 3:** Results of multiple regression analysis for BADS subscales.

	Dependent variables			
	BADS-AC	BADS-AR	BADS-WS	BADS-SI
**Predictor**				
PA-PL	1.09	−6.82*	−4.31	−3.53
**Covariates**				
Age	−0.36	−0.88	−0.39	−0.29
Sex	−0.20	–0.96	1.43	−0.18
BDI-II	−0.44*	0.99**	0.37**	0.36**
**Model statistics**				
R2	0.07	0.44	0.17	0.26
F	2.02	12.37**	3.94**	5.92**

*PA-PL* coefficients between physical activity and pleasure, *BDI-II* Beck Depression Inventory, *BADS* Behavioral Activation for Depression Scale, *BADS-AC* activation subscale, *BADS-AR* avoidance/rumination subscale, *BADS-WS* work/school impairment subscale, *BADS-SI* social impairment subscale.

**p < 0.01; *p < 0.05.

## Discussion

This report is the first of a study demonstrating the associations of physical activity amounts with pleasure and with behavioral characteristics in daily life by objectively measuring amounts of physical activity using an accelerometer and by assessing subjective pleasure based on reporting in an activity record.

Our results revealed that the physical activity amount, which is objectively measurable, is positively correlated with subjective pleasure in two-thirds of participants and negatively in one-third of them. Individual differences in the relations between physical activity and subjective pleasure were found. Furthermore, individuals who show more negative correlation between physical activity and pleasure, or who experience less pleasure from increased physical activity have a stronger tendency to show avoidance patterns. This effect was detected only when the severity of current depressive symptoms was controlled for. Therefore, the association between physical activity and pleasure can explain differences in avoidance behavior among people with the same severity of depression.

Earlier studies have pointed out that increased physical activity and mental health improvement do not simply correlate^1,4^. Those results have been assumed to be attributable to autonomous motivation intervention between the physical activity amount and mental health improvement^7^; the behavior being “fun” and “enjoyable” is an important factor in autonomous motivation^7^. A meta-analysis examining the relation between physical activity and mental health across different life domains in healthy adults was conducted by White et al^5^.They found that leisure-time and transport physical activities had a positive association with mental health, but work-related and household physical activities had no relation with mental health. Furthermore, Chu et al.^9^ used an accelerometer to explore the association of physical activity and mental health by measuring the amounts of physical activities in three domains: work, transport, and leisure-time. They found positive correlation only for leisure-time physical activity. Differences observed in the relation between physical activity and mental health across domains have been regarded as affected by the qualitative aspects of exercise including autonomous motivation and subjective pleasure, which have never been investigated directly. Furthermore, when measuring exercise by self-reporting, impairment by memory bias hindered detailed investigations of the association between the individual activity level and subjectively measured pleasure. To resolve that difficulty, we applied objective measurement of physical activity amount using accelerometers and subjective assessment of pleasure per hour, which led to a successful demonstration of the association between them.

Although more than two-thirds of healthy participants in our study showed a positive correlation between physical activity and pleasure from it, one-third of them showed a negative correlation, which suggests that there are individual differences in the correlation coefficient.

The BADS treats avoidance behavior and rumination as one factor because individuals use rumination as a strategy for avoidance of aversive situations and tasks^10^. Consequently, the BADS-AR measures a depressive "avoidance pattern". As many reports of earlier studies describe, this predicted frequent avoidance pattern, rumination/avoidance behavior, is related to depression. The calculation of a simple correlation coefficient also supported this argument. Furthermore, it has been pointed out that avoidance patterns deprive us of opportunities to obtain positive reinforcers in our lives^11^. Reports of earlier research have described a negative correlation between avoidance patterns and reward perception^12,13^. These findings suggest that when avoidance patterns predominate, it might be difficult to feel the pleasure that should accompany increased physical activity, even if the physical activity is rewarding. As described above, the two groups based on correlation between physical activity and pleasure might reflect differences in whether avoidance patterns interfere with the perception of reward. In this study, avoidance patterns were measured as usual behavioral tendencies using a questionnaire: the BADS. Integrating real-time detection of avoidance patterns, such as with an experience sampling method, are hoped to facilitate wider understanding of our results. This study demonstrated that behavioral characteristics of rumination/avoidance behaviors which produced less pleasure increased physical activity, but the effect was not associated with their amount of physical activity itself in the first week, which suggests that depressive behaviors are not attributable to the amount of physical activity, but to the subjective pleasure associated with the physical activity, i.e., the behavior contents. This was particularly characteristic among individuals with the same severity of depressive symptoms. Beyond the common view that depressive symptoms are associated with depressive avoidance patterns, a simple association between physical activity and pleasure might explain the individual differences in avoidance patterns.

For our study, all physical activities were quantified based on METs for analysis, but this unification method might affect accuracy. The intuitive mode of rating pleasure on a scale of 10 has not been evaluated for its consistency with other psychometric indexes to measure mental health. This study assessed the association between physical activity and pleasure in healthy university students. Therefore, it is unclear if the results are applicable to prevention of mental disorders or practical treatment of patients with mental disorders. Particularly, the BDI-II scores of participants were generally similar to or lower than those of university students participating in a larger study^14^ (5.92–7.85) and BADS subscale scores, the scores of AR and SI were slightly lower than those in earlier reports (AR = 14.16, SI = 3.42)^10^. The participants might have generally been healthier in terms of their mental status, although they were typical university students. Furthermore, given that the activities of university students vary considerably from person to person including study, part-time job, and club activities, similar studies should be conducted for adults whose activities are limited by their work and family situations.

When increased physical activity or physical intervention is planned to improve mental health in healthy people, merely increasing the amount of physical activity is insufficient: increasing experience of pleasure from it is fundamentally important. Monitoring functions of behavior in individuals and assuring activities that are accompanied with pleasure might be effective.

## Participants and methods

### Participants

The study participants were 66 students from Hiroshima University who had completed the measurements (42 men, 24 women; mean age of 21.7 ± 1.60). Descriptive statistics of participants ' BMI, IQ, and exercise history are presented in Supplementary Table S2.

### Study design

On the first day of the study, Day 1, after informed consent to participation in the study was obtained, accelerometers were distributed. Participants were instructed how to fill in the activity record. The participants wore an accelerometer for 7 days; on day 8, the accelerometer was returned and questionnaires on psychological indices were distributed, completed, and then collected. The activity record of 9 PM the preceding day to 9 PM on the day was submitted by e-mail from 9 PM to 0 AM. All participants who consented to join the study completed the measurement period without missing a beat. Because 1 MET corresponds to the energy cost of sitting quietly, the non-wear time was defined as the time when the 1-min MET output from the device became zero after the end of the measurement. The time period including the non-wear time was determined as the missing time when aggregating the hourly physical activity (describing in “Amount of physical activity”). Eight participants were excluded from the analysis because the missing time, including this non-wear time, exceeded 20% (33.6 h) of the 165 h.

### Measurements

#### Amount of physical activity

Using a wrist-worn tri-axial accelerometer device (UW-301BT Life Log; Hitachi Ltd., Tokyo), we measured metabolic equivalents (METs), an indicator of physical activity intensity, per minute from the resultant signal. Conversion from accelerations to METs was done using predictive equations^15,16^ implemented in the device. We took the METs directly as the output. Participants were instructed to wear the accelerometer continuously on the wrist of the nondominant arm, except during bathing. Because participants started wearing the device at different times of Day 1, data of 165 h in all from 0 AM on Day 2 to 9 PM on Day 8 were used for analyses. The total MET values per hour were calculated as the amount of physical activity for the hour. When 0 of METs per minute were included in the hour, we inferred that the hour included non-wearing time of the device. Therefore, the amount of physical activity for that hour was recorded as a missing value without calculation.

#### Pleasure from the activity record

During the experimentation period with the accelerometer, participants recorded their activities each hour in an activity record of electronic file and rated the amount of pleasure from each activity on a scale of 10. During the recording of pleasure, participants were asked to select one of the activity types simultaneously from the pre-defined activity categories to be able to rate it for the main activity of the hour. The activity categories were 19 areas based on earlier studies^17^: meals, dressing, commuting to school/work, school, housework, caring/nursing, child-care, shopping, going out other than commuting, television/radio/newspaper/magazine, hobbies, study/self-study, exercise, community/volunteer activities, social activities, outpatient treatment, sleep, relaxation. On the first day of the experiment, each participant recalled past activities and identified the activity as anchor points corresponding respectively to 0, 50, and 100 levels of pleasure. Subsequently, the participants were instructed to rate the intensity of their pleasure from 0 to 100 on a 10-point scale (10-point intervals) for each hourly activity during the week based on the anchors. To minimize failures in recording them, participants were instructed to send the table for the day by e-mail at a scheduled time (from PM 9 to AM 0) every day. Each participant selected one activity from 19 categories for every hour. The English version of the activity record in this study is presented in Supplementary Table S3.

#### Psychological indexes

Participants answered the following questionnaires on Day 8 when they finished the measurement of physical activity and had filled out the activity record.

i. Japanese version of the Beck Depression Inventory – Second Edition (BDI-II)^18^
  The BDI-II is the most widely used self-report inventory to measure depression severity. Scores of 0–63 and higher indicate a higher severity of depression^10^.
ii. Japanese version of Behavioral Activation for Depression Scale (BADS)^13^
  The BADS was invented to measure depression-related behaviors and dysfunction in the context of behavioral activation therapy^10^. It comprises four subscales of Activation (AC), Avoidance/Rumination (AR), Work/School Impairment (WS), and Social Impairment (SI). Its reliability and validity have been documented.

### Calculation of the index for physical activity—pleasure

To assess the association between the amount of hourly physical activities (PA) from the accelerometer and the hourly pleasure (PL) level, which is recorded subjectively with the activity record, the correlation coefficient between them for each hour was calculated for each person as an index for Physical Activity—Pleasure (PA-PL). Pleasure levels for the activity of sleeping in the activity record were all corrected to 0. Figure 1 shows the time-series plot of PA and PL of representative samples along with the value of their PA-PL index.

### Statistical analysis

To examine how the calculated PA-PL index affects individual behavior characteristics, we performed multiple linear regression analysis using the PA-PL index as the explanatory variable and the scores of BADS subscales as dependent variables. The scores of BDI-II, age, and gender were included as covariates for the analysis. All analyses were performed using “statsmodels” pakage (ver.0.11.0), a library for statistical analysis in Python.

### Ethical consideration

We conducted the study after providing all participants a written explanation describing the study purpose, the voluntary nature of participants’ participation, the confidentiality of the participants’ personal information, and the publication of the study results. All participants provided their written informed consent. This study protocol was approved by the Ethics Committee for Epidemiological Research of Hiroshima University and adhered to relevant ethical guidelines.

## Supplementary Information

Supplementary Information.
