# Supplementary material for: Decreased physical activity with subjective pleasure is associated with avoidance behaviors
Source: Sci Rep. 2022 Feb 18;12:2832. doi: 10.1038/s41598-022-06563-3 (PMC8857298; doi:10.1038/s41598-022-06563-3)
Supplement: Supplementary file 1 — Supplementary Information. [file 41598_2022_6563_MOESM1_ESM.docx]

**Supplementary information for “Decreased physical activity with subjective pleasure is associated with avoidance behaviors”**

Authors: Fumi Kagawa¹, Satoshi Yokoyama¹, Masahiro Takamura²³, Koki Takagaki⁴, Yuki Mitsuyama¹, Ayaka Shimizu¹, Ran Jinnin¹, Hirotaka Ihara¹, Akiko Kurata¹, Go Okada¹,Yasumasa Okamoto¹*

¹Department of Psychiatry and Neurosciences, Graduate School of Biomedical and Health Science, Hiroshima University, Hiroshima, Japan

²Department of Neurology, Shimane University, Shimane, Japan

³Brain, Mind and KANSEI Sciences Reserch Center, Hiroshima University, Hiroshima, Japan

⁴Health Service Center, Hiroshima University, Hiroshima, Japan

*Corresponding author: Yasumasa. Okamoto, M.D., Ph.D. (oy@hiroshima-u.ac.jp)

***Supplementary Information***

**Supplementary Results**

Table S1

**Supplementary Methods**

Table S2, Tables S3

**Supplementary Results**

**Table S1. Simple correlation coefficients with BADS subscale.**

|  | BADS-AC | | BADS-AR | | BADS-WS | | BADS-SI | | BDI-II | |
| --- | --- | --- | --- | --- | --- | --- | --- | --- | --- | --- |
|  | *r* | *p* | *r* | *p* | *r* | *p* | *r* | *p* | *r* | *p* |
| PA-PL | 0.04 | .77 | -0.22 | .10 | -0.21 | .12 | -0.23 | .08 | -0.10 | 0.94 |
| BDI-II | -0.35 | .01 | 0.64 | <.001 | 0.40 | .00 | 0.49 | <.001 | - | - |

BADS, Behavioral Activation for Depression Scale; BADS-AC, Activation subscale; BADS-AR, Avoidance/Rumination subscale; BADS-WS, Work/School Impairment subscale; BADS-SI, Social Impairment subscale

**Supplementary Methods**

**Table S2. Subjects' BMI, estimated IQ, and past exercise experience.**

|  |  | mean ± SD |
| --- | --- | --- |
| BMI |  | 20.90 ± 2.56 |
| Estimated IQ | | 109.52 ± 6.90 |
| Past exercise history (hours per week) | |  |
|  | Preschool | 0.98 ± 1.15 |
|  | 1st to 3rd grade of elementary school | 1.85 ± 2.54 |
|  | 4th to 6th grade of elementary school | 2.50 ± 3.67 |
|  | Junior high school | 7.44 ± 7.63 |
|  | High school student | 5.16 ± 6.74 |
| Note: Estimated IQ was calculated using the 25-item Japanese version of the NART (Matsuoka et al., 2002)^1^. For past exercise experience, participants were asked to retrospectively self-report their average exercise time per week at each period. | | |

**Supplementary Methods**

**Table S3. Activity record with assessment of pleasure.**

**[Activities that correspond to the scale of pleasure]**

| **0** | Ex. Lying around at home. |
| --- | --- |
| **50** | Ex. Work hard at club sports. |
| **100** | Ex. Set a new personal best. |

Note: These activities are determined by the individual reflecting on previous activities before beginning the recording of activities.

**[Activity record]**

|  |  | ↓ Select an anctivity |  |
| --- | --- | --- | --- |
| Date | time | activity type | Pleasure(0-100) |
| 8-May | 21:00 | Meals | 80 |
|  | 22:00 | School | 0 |
|  | 23:00 | Relaxation | 20 |
| 9-May | 0:00 | Relaxation | 20 |
|  | 1:00 | Sleep | 10 |
|  | 2:00 | Sleep | 10 |
|  | 3:00 |  |  |
|  | 4:00 |  |  |
|  | 5:00 |  |  |
|  | 6:00 |  |  |
|  | 7:00 |  |  |
|  | 8:00 |  |  |
|  |  |  |  |
|  | time | activity type | Pleasure(0-100) |
| 9-May | 9:00 |  |  |
|  | 10:00 |  |  |
|  | 11:00 |  |  |
|  | 12:00 |  |  |
|  | 13:00 |  |  |
|  | 14:00 |  |  |
|  | 15:00 |  |  |
|  | 16:00 |  |  |
|  | 17:00 |  |  |
|  | 18:00 |  |  |
|  | 19:00 |  |  |
|  | 20:00 |  |  |

**Please select one of the following categories for "Activity type".**

- meals
- dressing
- commuting to school/work
- School
- Housework
- Caring / Nursing
- child-care
- Shopping
- going out other than commuting
- television / radio / newspaper / magazine
- hobbies
- study / self-study
- exercise
- community / volunteer activities
- social activities
- outpatient treatment
- sleep
- relaxation

References

1. Matsuoka, K. et al., Development of Japanese Adult Reading Test (JART) for Predicting Premorbid IQ in Mild Dementia. *Seishin Igaku*44,5,503-511(2002).
